# Supplementary material for: Identification of human D lactate dehydrogenase deficiency
Source: Nat Commun. 2019 Apr 1;10:1477. doi: 10.1038/s41467-019-09458-6 (PMC6443703; doi:10.1038/s41467-019-09458-6)
Supplement: Supplementary file 1 — Supplementary Information [file 41467_2019_9458_MOESM1_ESM.docx]

**Identification of human D lactate dehydrogenase deficiency**

*Monroe et al*

**Supplementary Table 1: Page 2**

**Supplementary Table 2: Page 3**

**Supplementary Figure 1: Page 4**

**Supplementary Table 3: Page 5**

**Supplementary Table 1:** **Regions of homozygosity in the patient greater than 1 Mb**. Genome build GRCh37/hg19.

| **Chromosome** | **Start Position** | **End Position** | **Size (basepair)** |
| --- | --- | --- | --- |
| 13 | 85802402 | 98498677 | 12696275 |
| 11 | 29651299 | 40817882 | 11166583 |
| 12 | 34512767 | 38651375 | 4138608 |
| 17 | 21770128 | 25568707 | 3798579 |
| 16 | 74873927 | 78363380 | 3489453 |
| 5 | 71489305 | 74242852 | 2753547 |
| 10 | 45556652 | 47593133 | 2036481 |
| 2 | 197129767 | 198962243 | 1832476 |
| 6 | 62415600 | 63936511 | 1520911 |
| 15 | 43344886 | 44847415 | 1502529 |
| 3 | 74479445 | 75974251 | 1494806 |
| 1 | 28211650 | 29665936 | 1454286 |
| 19 | 42001210 | 43372386 | 1371176 |
| 2 | 116014077 | 117364078 | 1350001 |
| 5 | 139188856 | 140519095 | 1330239 |
| 4 | 97935617 | 99214851 | 1279234 |
| 2 | 186218867 | 187481835 | 1262968 |
| 7 | 73909796 | 75166580 | 1256784 |
| 14 | 105904830 | 107146692 | 1241862 |
| 8 | 50265730 | 51469405 | 1203675 |
| 18 | 39796976 | 40996805 | 1199829 |
| 5 | 36716862 | 37892952 | 1176090 |
| 6 | 145800915 | 146972068 | 1171153 |
| 7 | 97136298 | 98233514 | 1097216 |
| 11 | 112040136 | 113133676 | 1093540 |
| 6 | 114860722 | 115944735 | 1084013 |
| 3 | 81988359 | 83039277 | 1050918 |
| 2 | 97595991 | 98629966 | 1033975 |
| 4 | 110000675 | 111030787 | 1030112 |
| 8 | 85474803 | 86493592 | 1018789 |

**Supplementary Table 2:** **Genes present in the regions of homozygosity of the patient that are greater than 1 MB.** Genome build GRCh37/hg19.

| **Chromosome** | **Start position** | **Stop position** | **Gene** |
| --- | --- | --- | --- |
| 16 | 74907468 | 75034071 | WDR59 |
| 16 | 75032928 | 75144892 | ZNRF1 |
| 16 | 75145758 | 75150669 | LDHD |
| 16 | 75182390 | 75206134 | ZFP1 |
| 16 | 75237994 | 75241083 | CTRB2 |
| 16 | 75252898 | 75258822 | CTRB1 |
| 16 | 75262928 | 75301951 | BCAR1 |
| 16 | 75327596 | 75467383 | CFDP1 |
| 16 | 75446582 | 75498604 | RP11-77K12.1 |
| 16 | 75476952 | 75499395 | TMEM170A |
| 16 | 75510949 | 75529282 | CHST6 |
| 16 | 75562430 | 75579326 | RP11-77K12.7 |
| 16 | 75562433 | 75569145 | CHST5 |
| 16 | 75572015 | 75590176 | TMEM231 |
| 16 | 75600249 | 75611779 | GABARAPL2 |
| 16 | 75630879 | 75657198 | ADAT1 |
| 16 | 75661622 | 75682541 | KARS |
| 16 | 75681684 | 75795051 | TERF2IP |
| 16 | 75728367 | 75734089 | AC025287.1 |
| 16 | 76311176 | 76593135 | CNTNAP4 |
| 16 | 76587314 | 76669520 | RP11-58C22.1 |
| 16 | 77224732 | 77236302 | MON1B |
| 16 | 77233294 | 77247112 | SYCE1L |
| 16 | 77281710 | 77469011 | ADAMTS18 |
| 16 | 77756411 | 77776157 | NUDT7 |
| 16 | 77822427 | 78014004 | VAT1L |
| 16 | 78056412 | 78100658 | CLEC3A |
| 16 | 78133310 | 79246564 | WWOX |


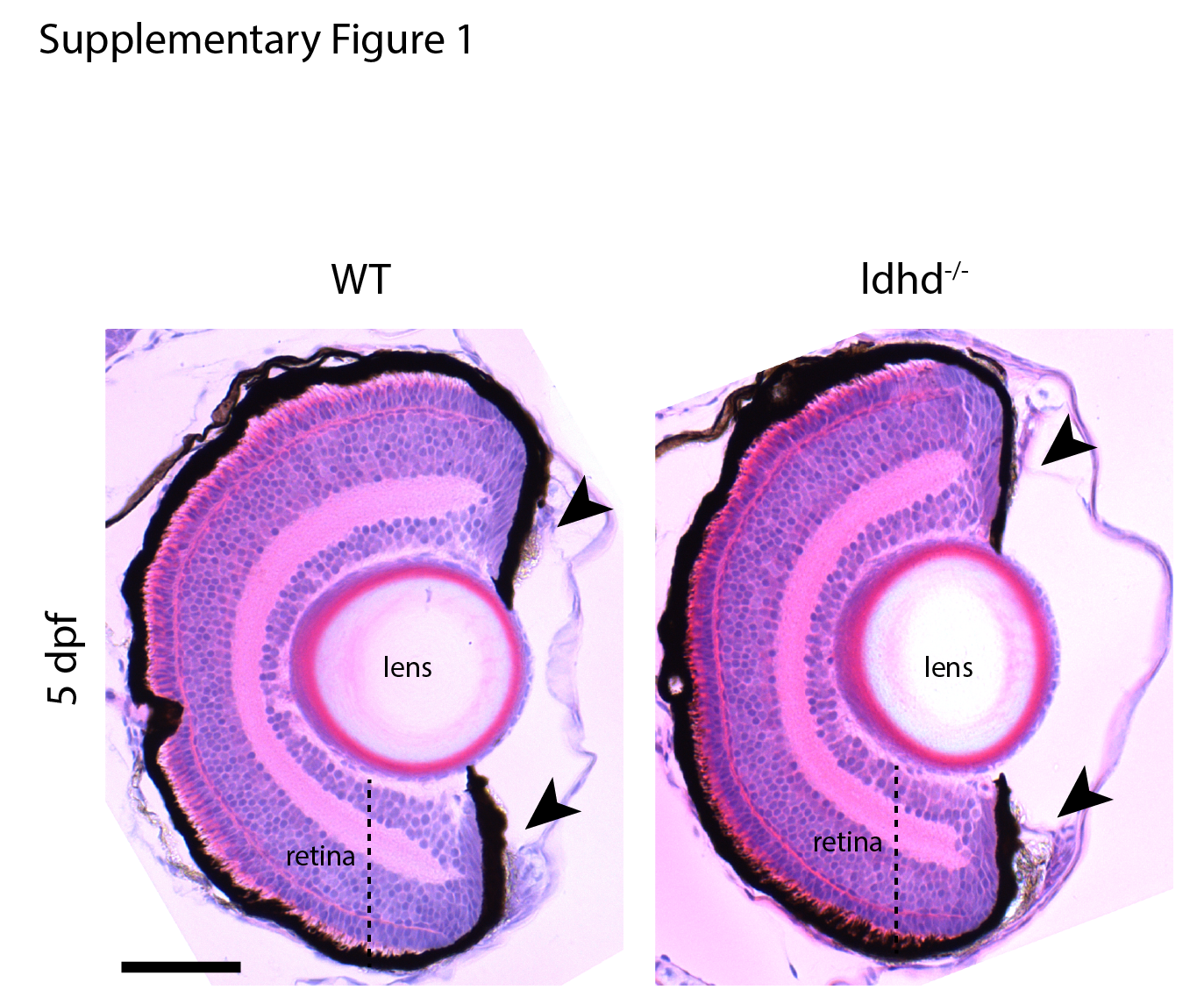


**Supplementary Figure 1:** ***MZ ldhd^-/-^* embryos show no defective developing iris.** Comparison of H&E stained sections of Wildtype (left panel) and *MZ ldhd^-/-^* (right panel) 5dpf embryos reveals no defects in the developing iris (arrowheads). Scale bar: 50 μm.

**Supplemental Table 3:** **Primer sequences for Sanger sequencing of LDHD exonic and intron/exon splice regions.**

| **Primer name** | **Sequence** |
| --- | --- |
| LDHD_1F | TGAACCTCGCCTCTCCTTTA |
| LDHD_2F | TGGAAAAGAGGCATTTCTGG |
| LDHD_3F | CTCGCTTGCTGTAGCCTCA |
| LDHD_4F | GATGGTCCCAGCACACCTT |
| LDHD_5F | GGTTCAGGGGACCTTCCTC |
| LDHD_6F | TTGTGGAGCAGAAAGCACTG |
| LDHD_7F | CATTGGTGAGCTGAGGATGG |
| LDHD_8F | AGGTATGCTGGGGTGGAGT |
| LDHD_9F | GACCCATCCTCAGCCTTGA |
| LDHD_10F | GAGAGGTGGGGAGGTGTCA |
| LDHD_11F | CTGCTGCAGGAGGAGGTG |
| LDHD_12F | CAGCGAGCCCACTGTATCTG |
| LDHD_1R | CCAAGGTCAGGTCACTTGGT |
| LDHD_2R | TGAGTGAGGAGGAAGGCAAC |
| LDHD_3R | ATTCGGTCCATATGCGTCA |
| LDHD_4R | CGTACAGAAGGCAGCCTCA |
| LDHD_5R | GAAGCCTAGCCTGCCAAAC |
| LDHD_6R | CAGGCATCCATCATGACTTC |
| LDHD_7R | GTGCCGTGCTGTCCAAAG |
| LDHD_8R | CTGCAGCCCTTGTTTCTAGC |
| LDHD_9R | GTTCTGCAAAAGCCTTGACC |
| LDHD_10R | GTCAGGGAACTTGTGGGCTA |
| LDHD_11R | ACCAGGTGAAGGGGGAAG |
| LDHD_12R | CCTCCTCCCCAGGCTATAAG |
